# Cerebrospinal Fluid Cutaneous Fistula Following Neuraxial Anesthesia for Cesarean Delivery

**DOI:** 10.7759/cureus.32895

**Published:** 2022-12-24

**Authors:** Marta Sanha, Inês Vaz, Helena Barbosa, Susana Alves, Manuela Paiva

**Affiliations:** 1 Anesthesiology, Centro Hospitalar Vila Nova de Gaia/Espinho, Porto, PRT

**Keywords:** caesarian sections(c/s), epidural, obstetric anaesthesia & analgesia, spinal anaesthesia, cerebrospinal fluid-fistula

## Abstract

Cerebrospinal fluid (CSF) cutaneous fistula is an unusual but potentially serious complication of neuraxial procedures. While combined spinal-epidural (CSE) technique or spinal/epidural techniques alone are standard in obstetric anesthesia, subsequent persistent CSF leak is rarely reported in the obstetric population. Clinical presentation ranges from asymptomatic states and only abnormal leakage through the puncture site to severe cases with meningitis or subdural hematoma. Both conservative and invasive approaches are suitable for management, but no formal guidelines on how to diagnose and manage this condition are available, and hence clinicians have to rely on their experience. We present a case of a 35-year-old parturient scheduled for an elective cesarean delivery with a persistent CSF leak three days after epidural catheter removal. The leakage was managed with both suturing of the skin site and conservative methods such as hydration, bed rest, and oral analgesics, with no adverse effects for the patient.

## Introduction

Neuraxial techniques are considered the gold standard in obstetric anesthesia, for both labor analgesia and as an anesthetic technique for cesarean delivery [1]. The combined spinal-epidural (CSE) technique is often employed, as it enables a rapid onset of anesthesia with the possibility to maintain both analgesia and anesthesia through the catheter [2]. Although it is commonly used, complications still occur. Cerebrospinal fluid (CSF) cutaneous fistula is a rare but potential hazard of the neuraxial blockade, as it may complicate the procedure with meningitis or pseudomeningocele [3]. It is defined as an abnormal leakage of CSF from subarachnoid space to the skin, through the epidural insertion site. The estimated incidence of this complication is 0.16% [3]. It usually becomes evident after the catheter removal. Clinical presentation varies from asymptomatic cases and those with post-dural-puncture headaches with nausea, vomiting, and photophobia to severe cases with meningitis or subdural hematoma [4]. The symptoms are accompanied by leakage of the fluid through the puncture site, necessitating referral to anesthetic teams. There are scarce data on this condition in the literature, and both conservative and more invasive strategies can be adopted for its management [5]. Nevertheless, guidelines on how to diagnose and manage this condition are limited [4] and the best clinical approach is yet to be determined. We report a case of persistent CSF leakage in a 35-year-old parturient after an uneventful CSE anesthesia for an elective cesarean delivery.

## Case presentation

A 35-year-old parturient, nullipara, with 39 weeks of estimated gestational age was scheduled for a cesarean delivery due to breech presentation. There was no relevant medical history, and she had a body mass index of 25 kg/m^2^. Laboratory analysis revealed no abnormalities. As there was no contraindication to the neuraxial approach, the anesthetic plan adopted was a CSE technique. After explaining the anesthetic procedure to the patient, written informed consent was obtained to perform the technique. An uneventful CSE technique was performed with the patient in a sitting position at the first attempt. Subcutaneous injection of 2 mL of 2% Lidocaine was first administered at L3-L4 interspace, followed by the puncture with a CSE kit containing an 88-mm 18G Tuohy needle, a 138.5-mm 27G spinal needle, and an epidural catheter; 8 mg of hyperbaric bupivacaine with 2.5 mcg of sufentanil were administered in the subarachnoid space and a Braun PERIFIX® SoftTip Epidural Catheter was inserted 4 cm in cephalic orientation into the epidural space. The surgical procedure was completed uneventfully. The epidural catheter was left in situ for 24 hours postpartum for analgesic purposes and then removed without apparent complication.

**Figure 1:**
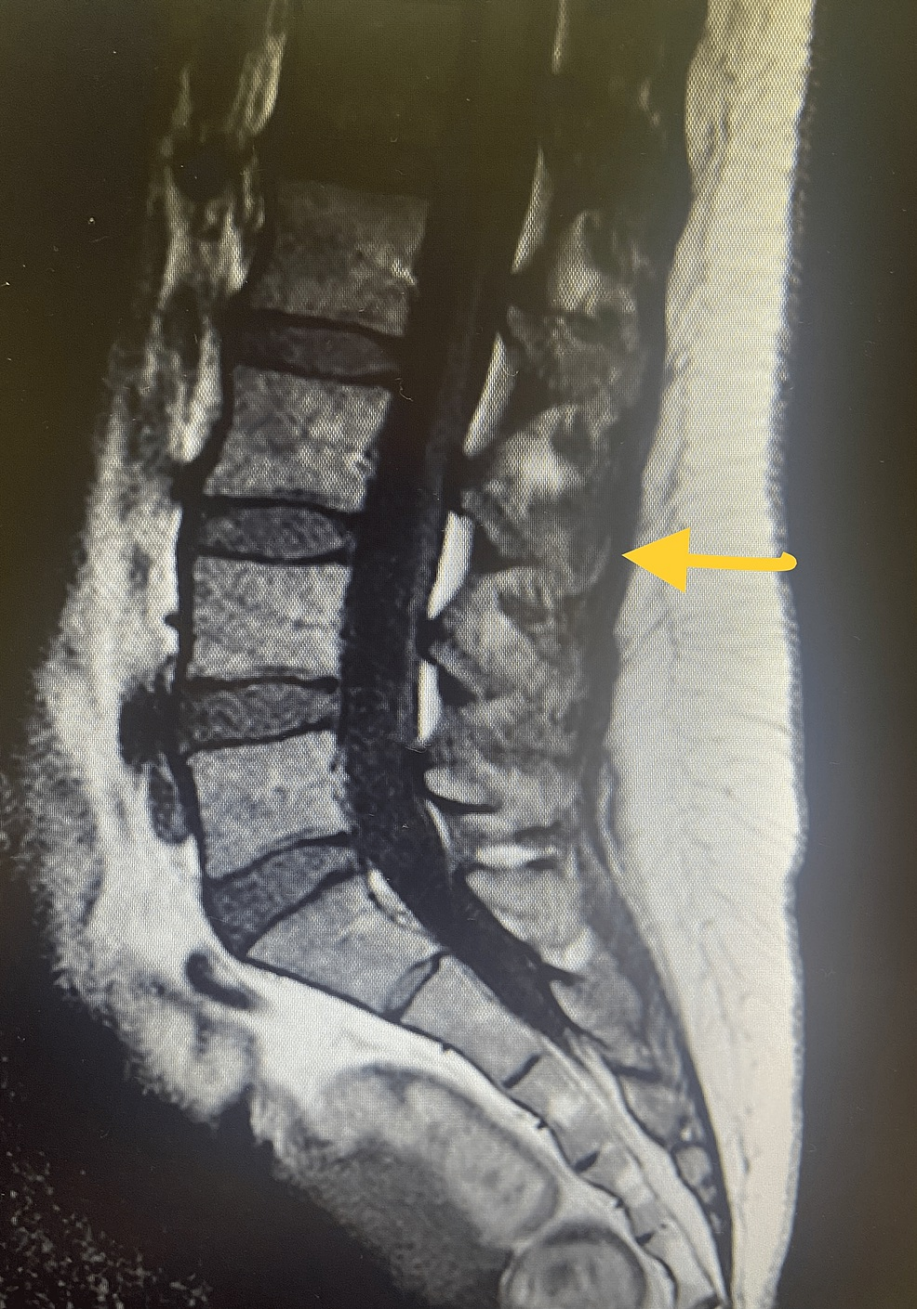
Lumbosacral spine MRI No abnormalities were detected on MRI. The yellow arrow points to the puncture site MRI: magnetic resonance imaging

On day three postpartum, clear fluid drainage from the catheter insertion site was noted, which prompted a referral to the anesthetic team. A quick Combur-Test® confirmed the presence of glucose, and the drainage was identified as CSF. Despite the noticeable leakage, the patient remained asymptomatic, denying any neurological symptoms including headache, nausea, nuchal rigidity, photophobia, or fever. The case was discussed with neurosurgery, and a diagnosis of CSF cutaneous fistula was made. The epidural insertion site was sutured, and hydration with bed rest was recommended. No more leakage was verified. The following day, the patient reported a headache, which was managed with oral analgesics. The CSF cutaneous fistula was effectively treated with both sutures at the epidural site and conservative measures. The patient was discharged on the seventh day postpartum, without encountering any other events. A lumbosacral spine MRI was performed a few months after the event to search for neuraxial anomalies or detect long-term complications. Neither congenital abnormalities, such as meningocele, nor any intracanalar collections were detected (Figure 1).

## Discussion

Very few cases of CSF cutaneous fistula following anesthetic techniques have been reported in the literature. However, most of these cases do not involve the obstetric population [5]. Whenever clear fluid is detected draining through the epidural insertion site, a differential diagnosis between CSF, interstitial fluid, or local anesthetic is required. The technique itself, besides the timing and clinical presentation, should be evaluated. Despite the low sensitivity of tests for glucose and low protein levels in the fluid, they are nevertheless quick, feasible, and clinically useful. Testing for the presence of beta-2 transferrin would be a more specific and sensitive approach, as it is a protein found only in the CSF and perilymph [6]. However, this is a time-consuming and expensive method and, therefore, not always requested [4-7]. Due to the rarity of this clinical situation, there is no standardized treatment or formal recommendations. The successful management of this complication ranges from conservative measures to more invasive approaches including cutaneous stitching or even epidural blood patch. The conservative treatment could be an effective approach for asymptomatic patients or if contraindications for invasive procedures are present [8]. Any anesthetic technique involving dural puncture may lead to CSF leakage. Hence, close monitoring of the patients after the neuraxial approach is of utmost importance for prompt detection. Although some studies have reported the use of antibiotic prophylaxis to prevent meningitis, there is scant evidence to support this and it should not be encouraged, as it contributes to the resistance of microbes to antimicrobial agents, a major problem faced by medical practitioners these days [9]. In this particular case, the patient was asymptomatic, and no neurological signs were present. Hence, after a multidisciplinary discussion involving both anesthetic and neurosurgery teams, the decision to perform a cutaneous stitch was made, in order to promote tract closure. Conservative measures such as hydration, bed rest, and oral analgesics such as aspirin were also provided.

## Conclusions

Since there is sparse data on CSF cutaneous fistula in the literature and no optimal treatment method has been devised for the condition yet, we usually rely on clinical experience to guide us in its management. Although it is often asymptomatic or manifests only minor symptoms, anesthetic and obstetric teams must be aware of this rare but possible complication of such frequent techniques in the obstetric population. This case supports and reinforces the efficacy of the application of both conservative and minimally invasive measures as a reliable treatment method for CSF cutaneous fistula after CSE anesthesia, which helps to avoid the potential complications of more invasive options. Clinical experience is an important factor in managing these cases since no formal evidence-based recommendations are available.
